# Compensatory Hypertrophy in Paediatric Patients with a Unilateral Ureteropelvic Junction Obstruction

**DOI:** 10.1016/j.euros.2021.09.008

**Published:** 2021-10-27

**Authors:** Sander Groen in 't Woud, Nieke Reuver, Wout F.J. Feitz, Josine S.L.T. Quaedackers, Rien Nijman, Martijn Steffens, Liesbeth L.L. de Wall, Nel Roeleveld, Michiel F. Schreuder, Loes F.M. van der Zanden

**Affiliations:** a Radboud university medical center, Radboud Institute for Health Sciences, Department for Health Evidence, Nijmegen, The Netherlands; b Radboud university medical center, Amalia Children’s Hospital, Radboud Institute for Molecular Life Sciences, Department of Pediatric Nephrology, Nijmegen, The Netherlands; c Radboud university medical center, Amalia Children’s Hospital, Radboud Institute for Molecular Life Sciences, Division of Pediatric Urology, Department of Urology, Nijmegen, The Netherlands; d University Medical Center Groningen, Department of Urology, Groningen, The Netherlands; e Isala Hospital, Department of Urology, Zwolle, The Netherlands

**Keywords:** Urogenital abnormalities, Ureteral obstruction, Chronic kidney disease

## Abstract

**Background:**

Compensatory hypertrophy is common in children with solitary functioning kidney, but it is unknown whether it also develops in children with unilateral partial reduction of kidney function.

**Objective:**

The aim of this study was to assess whether children with a unilateral ureteropelvic junction obstruction (UPJO) show compensatory growth of the unaffected kidney. Furthermore, we investigated whether the length of the unaffected kidney was related to the degree of split kidney function lost and other possible risk factors. Lastly, we studied a possible relationship with signs of kidney injury.

**Design, setting, and participants:**

We retrospectively analysed clinical information from 194 children with a unilateral UPJO who participated in the Aetiologic research into Genetic and Occupational/environmental Risk factors for Anomalies in children (AGORA) data- and biobank. Data on kidney length, split kidney function, and other factors possibly associated with kidney length were extracted from electronic patient records.

**Outcome measurements and statistical analysis:**

Pearson’s correlation coefficients between the split kidney function and unaffected kidney length were calculated. Multivariable logistic regression analyses were performed to identify factors associated with kidney length and signs of kidney injury.

**Results and limitations:**

Most children with a UPJO had an unaffected kidney length above the reference for age at the end of follow-up (median age 6.5 yr). A correlation with split kidney function was present only in children with a split kidney function of ≥60% in the unaffected kidney (*r* = 0.41). Aside from split kidney function, UPJO side was the only determinant of kidney length, while no associations between kidney length and kidney injury were identified.

**Conclusions:**

Compensatory growth was visible in most children with a UPJO after sufficient follow-up time and was correlated with split kidney function in children with a severe UPJO. Contralateral kidney length provided no clear prognostic value for developing kidney injury. Studies with more patients and additional biomarkers of kidney injury are needed to further personalise care.

**Patient summary:**

Children with obstruction of urine outflow in one kidney often had a larger contralateral kidney. However, the size of this kidney could not be used to predict which children would develop kidney injury.

## Take Home Message

Compensatory hypertrophy is present in most children with unilateral ureteropelvic junction obstruction after sufficient follow-up time. Contralateral kidney length provided no clear prognostic value for developing kidney injury. Studies with more patients and additional biomarkers of kidney injury are needed to personalise care further.

## 1 Introduction

Kidney mass reduction will generally result in a compensatory response of the remaining kidney tissue, both functional and structural. If the kidney mass reduction occurs in one kidney, the contralateral kidney will enlarge—a process that is often described as compensatory hypertrophy. Except for an increase in nephron size (hypertrophy), however, formation of additional nephrons (hyperplasia) may also underlie the observed growth in kidney size. The type of enlargement depends on the timing of the reduction in kidney mass, since hyperplasia is possible only during on-going nephrogenesis (ie, before term birth) [1], [2].

The exact mechanisms underlying compensatory hypertrophy are largely unknown. Activation of the mTORC pathway by growth factors such as insulin-like growth factors 1 and 2 or through production of nitric oxide may stimulate compensatory hypertrophy, but other mechanisms still need to be unravelled [3], [4]. Kidney hypertrophy is seen in patients with a congenital solitary functioning kidney, after donor nephrectomy, and in adults undergoing partial nephrectomy. In the latter situation, the magnitude of hypertrophy is proportional to the degree of kidney mass reduction [4], [5], [6], [7], [8]. Whether a partial reduction in kidney mass in children also results in an increase in the size of the contralateral kidney is unknown, as is its proportionality to the amount of kidney tissue that is lost. Even though partial nephrectomy is not performed commonly in children, various degrees of partial reduction in kidney function are frequently seen in children with a ureteropelvic junction obstruction (UPJO).

To gain more insight into the occurrence and clinical implications of compensatory kidney hypertrophy in children, we studied its prevalence, its proportionality to the amount of function decline, and the influence of other clinical factors on its occurrence in children with a unilateral UPJO. Furthermore, we set out to investigate whether the size of the contralateral or unaffected kidney was associated with signs of kidney injury later in life.

## 2 Patients and methods

### 2.1 Participants

From 2004 onwards, patients with congenital anomalies of the kidney and urinary tract who visited the paediatric urology department of the Radboud University Medical Center were asked to participate in the Aetiologic research into Genetic and Occupational/environmental Risk factors for Anomalies in children (AGORA) data- and biobank [9]. Parents of participating patients filled out a questionnaire and gave informed consent to access the medical files of their children. In 2016 and 2017, patients with obstructive uropathies who visited the University Medical Center Groningen and Isala Hospital Zwolle in 2002 or later, or who visited the Radboud University Medical Center in 1981 or later and had not yet participated, were approached to participate in AGORA retrospectively. The Medical Ethical Committee Arnhem-Nijmegen approved these studies. From the AGORA data- and biobank, all patients with a UPJO who had undergone a pyeloplasty before the age of 18 yr were selected for this study. Since we intended to study compensatory mechanisms in the unaffected kidney, we excluded patients with conditions that could affect the development of this kidney, such as bilateral UPJO, posterior urethral valves, or grade III or IV hydronephrosis in the unaffected kidney. We also excluded patients with cortex abnormalities of the unaffected kidney on ultrasound and/or renography.

### 2.2 Measurements

Information about the length of the unaffected kidney in its longest dimension was retrieved from electronic patient files. Only ultrasound scans in which no dilatation in the unaffected kidney was observed were used. Reference values for normal kidney length were adapted from the study of Rosenbaum et al. [10]. We calculated age-corrected kidney lengths by dividing the measured length of the unaffected kidney by the reference length for age.

To evaluate the degree of kidney function reduction due to UPJO, the relative contribution of the unaffected kidney to the total kidney function (split kidney function) was calculated from MAG-3 renographies. When multiple MAG-3 scans were made, the first one was used to define the function at presentation and the last one to define the split kidney function at the end of follow-up.

In addition to split kidney function, we assessed whether other factors were associated with the length of the unaffected kidney at the end of follow-up. We considered the following factors for these analyses: gender, age at diagnosis, side of UPJO, prematurity (birth before 37 wk of gestation), birth weight, and maternal medication use during pregnancy. Information on these variables was collected from medical files and maternal questionnaires. We considered asthmatic medication, antihypertensives, antiepileptic drugs, and antidiabetics (including insulin) as medication classes potentially affecting kidney development.

Information on kidney injury during follow-up was collected from patient files. We considered high blood pressure, use of antihypertensive medication, proteinuria, estimated glomerular filtration rate (eGFR) of <60 ml/min/1.73 m^2^, and/or necessity of kidney replacement therapy as a sign of kidney injury. High blood pressure was defined as a systolic and/or diastolic blood pressure above the 95th percentile for age and height in children, and above 140 mmHg (systolic) and/or 90 mmHg (diastolic) in adults [11]. Proteinuria was defined as a protein/creatinine ratio of >0.55 g/10 mmol in children aged between 6 and 24 mo and >0.22 g/10 mmol in older children [12]. The eGFR was estimated from serum creatinine values using the Bedside Schwartz formula for children between 3 mo and 18 yr of age and the Chronic Kidney Disease Epidemiology Collaboration (CKD-EPI) formula in adults [13], [14].

### 2.3 Statistical analysis

Spearman correlation coefficients (*r*_s_) between split kidney function and age-corrected length of the unaffected kidney were calculated at initial presentation (if both function and length were measured before pyeloplasty) and at the end of follow-up. We used 1000 bootstrap samples to calculate bias-corrected accelerated 95% confidence intervals (CIs) for the correlation coefficients. A priori, we hypothesised that a correlation between split kidney function and kidney length could also be nonlinear, for example, more pronounced in children with a severe reduction of function in the kidney with a UPJO. To visualise such correlations, we fitted locally estimated scatterplot smoothing (LOESS) lines in the scatterplots [15].

We investigated other factors possibly associated with the length of the unaffected kidney by comparing patients in the lowest quartile of age-corrected kidney length at the end of follow-up with those in the highest quartile. Crude (using only one determinant of interest) and adjusted (using all factors as determinants) odds ratios (ORs) with 95% CIs were estimated using binary logistic regression models. This multivariable regression analysis was performed on an imputed dataset with ten imputation sets to account for missing data. Identical methods were used to calculate associations with the signs of kidney injury.

Some patients were diagnosed with a UPJO in the first months of life based on antenatal screening, while others were diagnosed after a symptomatic presentation later in life. As these groups could reflect patients with different mechanisms and duration of disease, we performed stratified analyses based on the age of the first ultrasound (within 1 yr of birth or later). All analyses were performed using SPSS version 25, and all *p* values of <0.05 were considered to be statistically significant.

## 3 Results

Information on 318 children who had undergone a pyeloplasty before the age of 18 yr was retrieved from the AGORA data- and biobank. From these children, 67 were excluded because of possible contralateral kidney anomalies and 57 were excluded because no kidney length measurement was recorded. Of the 194 patients included in the analyses, kidney length at presentation was available for 96 children and kidney length at the end of follow-up was available for 127. Table 1 shows the demographic and clinical characteristics of the patients. Patients who presented after the age of 1 yr were more likely to have an enlarged unaffected kidney, but no other differences were observed between children who presented during or after the 1st year of life.

**Table 1:** Demographic and clinical characteristics of included children (*n* = 194)

Characteristic	Overall (*n* = 194)	Diagnosed <1 yr (*n* = 93)	Diagnosed >1 yr (*n* = 101)	*p* value
Female	53 (27)	24 (26)	29 (29)	0.65
Right-sided UPJO	64 (33)	33 (36)	31 (31)	0.48
Prematurity	14 (7.7)	8 (8.8)	6 (6.6)	0.58
Birth weight (g)	3450 (3120–3830)	3456 (3104–3940)	3440 (3120–3700)	0.32
Maternal medication use	11 (5.7)	6 (6.5)	5 (5.0)	0.65
Age at first ultrasound (yr)	0.54 (0.03–4.7)	0.12 (0.02–0.58)	7.2 (5.3–9.0)	
Length of the unaffected kidney at presentation (mm)	61 (50–83)	55 (47–62)	86 (83–95)	
Normalised length of the unaffected kidney at presentation	1.00 (0.94–1.06)	0.98 (0.94–1.06)	1.04 (0.96–1.08)	0.14
Length of the unaffected kidney above reference at presentation a	47 (49)	27 (40)	20 (69)	**0.01**
Age at first MAG-3 scan (yr)	0.75 (0.12–6.9)	0.12 (0.09–0.27)	6.9 (4.9–9.3)	
Split kidney function of the unaffected kidney at presentation (%)	55 (50–64)	54 (50–63)	56 (52–64)	0.07
Age at last ultrasound (yr)	6.5 (3.7–11)	3.5 (1.8–4.2)	11 (7.9–13)	
Length of the unaffected kidney at last ultrasound (mm)	89 (75–100)	74 (68–84)	98 (89–110)	
Normalised length of the unaffected kidney at last ultrasound	1.04 (0.97–1.11)	1.02 (0.95–1.09)	1.05 (1.01–1.12)	**0.04**
Length of the unaffected kidney above reference at last ultrasound a	85 (67)	30 (56)	55 (75)	**0.02**
Age at last MAG-3 scan (yr)	4.9 (2.0–9.5)	2.0 (1.3–3.4)	9.3 (6.2–12)	
Split kidney function of the unaffected kidney at last MAG-3 scan (%)	56 (51–65)	56 (51–63)	56 (51–65)	0.55
Signs of kidney injury b	68 (35)	30 (32)	38 (38)	0.43
Decreased eGFR	12 (6.2)	9 (9.7)	3 (3.0)	0.05
High blood pressure	43 (22)	15 (16)	28 (28)	0.05
Proteinuria	9 (4.6)	4 (4.3)	5 (5.0)	0.83
Use of antihypertensive medication	16 (8.2)	7 (7.5)	9 (8.9)	0.73

eGFR = estimated glomerular filtration rate; UPJO = ureteropelvic junction obstruction.

Data are presented as median (interquartile range) or *n* (%).

P-values in bold were statistically significant.

a Calculated using age-specific reference values from the study of Rosenbaum et al [10]. Kidney length at presentation was available in 96 children and kidney length at the end of follow-up was available in 127.

b The overall number of patients with any sign of kidney injury is lower than the sum of all different signs since some patients had more than one sign of kidney injury.

**Fig. 1:**
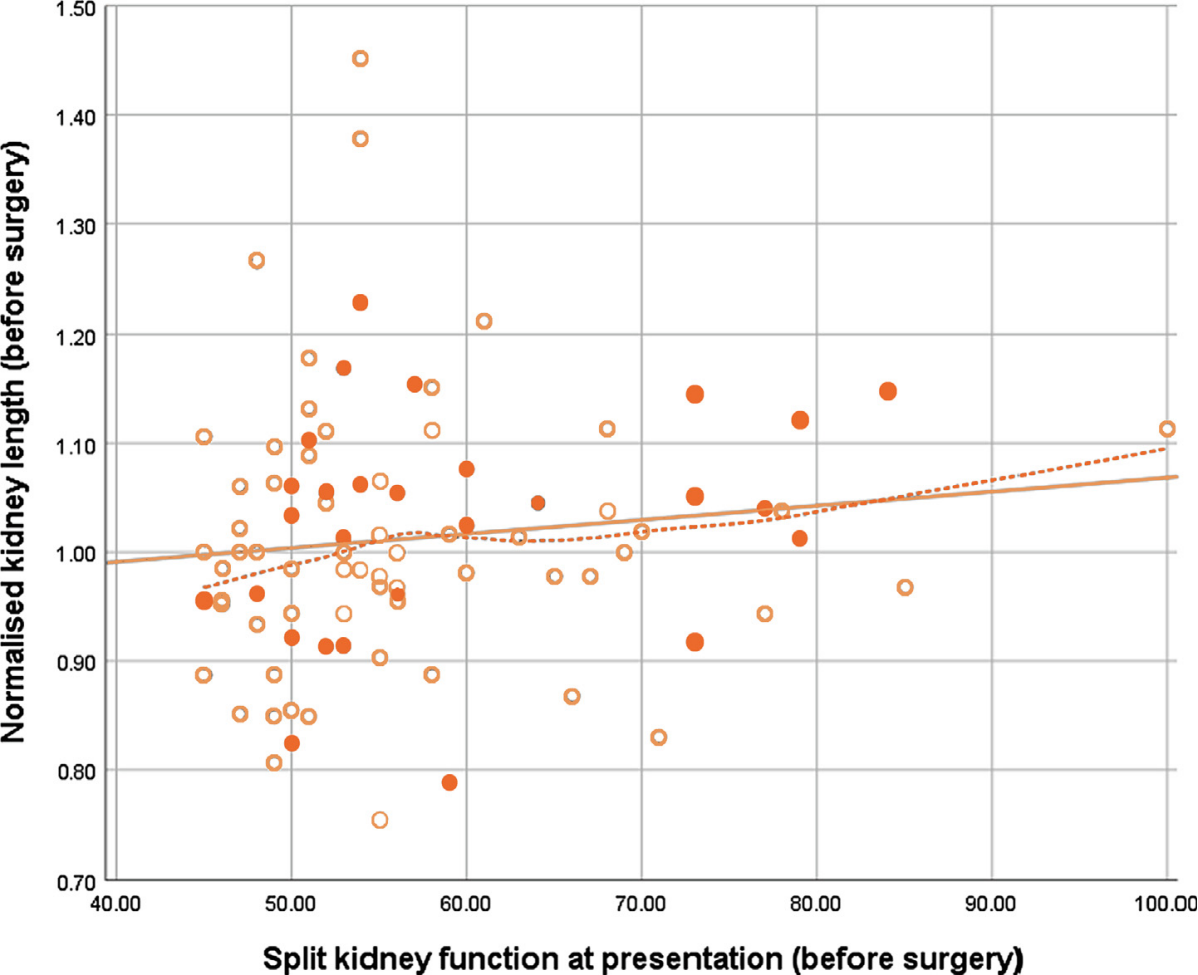
Scatter plot of age-corrected length of the unaffected kidney plotted against the split kidney function of the unaffected kidney at presentation. Open markers represent children diagnosed during the 1st year of life, and closed markers represent children diagnosed after the 1st year. The solid line represents the linear fit line; the dotted line was fitted with LOESS. LOESS = locally estimated scatterplot smoothing.

**Fig. 2:**
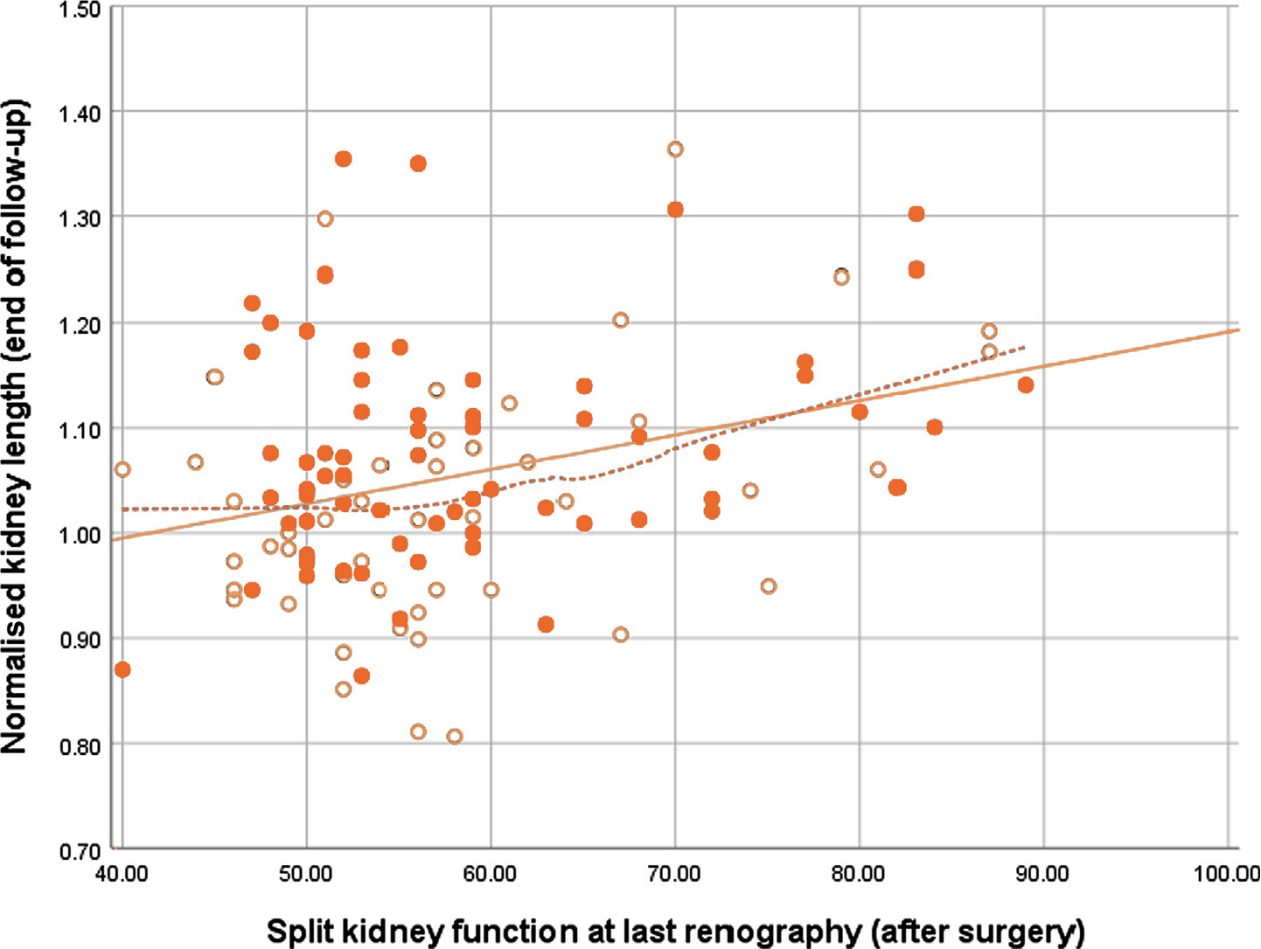
Scatter plot of age-corrected length of the unaffected kidney plotted against the split kidney function of the unaffected kidney at the end of follow-up. Open markers represent children diagnosed during the 1st year of life and closed markers represent children diagnosed after the 1st year. The solid line represents the linear fit line; the dotted line was fitted with LOESS. LOESS = locally estimated scatterplot smoothing.

At presentation, only a very weak correlation (*r*_s_ = 0.18) was observed between the split kidney function and the length of the unaffected kidney, which was stronger at the end of follow-up up (*r*_s_ = 0.30), especially for children diagnosed within the 1st year of life (Table 2, and Fig. 1, Fig. 2). Since the LOESS regression lines seemed to support our hypothesis of a stronger correlation in children with a more severely affected split kidney function, the correlations were also stratified for split kidney function (<60% vs ≥60%). A moderate correlation (*r*_s_ *=* 0.47) between kidney function and size was present in the group with ≥60% function at the end of follow-up, whereas no correlation was seen in the group with <60% function (Table 3).

**Table 2:** Correlations between split kidney function and normalised length of the unaffected kidney, stratified for diagnosis within or after the 1st year

	*n*	*r*_s_	95% CI
*At presentation*			
Overall	92 a	0.18	(–0.01 to 0.37)
Diagnosed <1 yr	64	0.09	(–0.19 to 0.35)
Diagnosed >1 yr	28	0.30	(–0.08 to 0.63)
*At end of follow-up*			
Overall	115 b	0.27	(0.08–0.44)
Diagnosed <1 yr	48	0.29	(–0.03 to 0.52)
Diagnosed >1 yr	67	0.22	(–0.04 to 0.44)

CI = confidence interval; *n* = number; *r*_s_ = Spearman correlation coefficient.

a The correlation at presentation could not be calculated for four patients due to missing results of the first MAG-3 scan.

b The correlation at the end of follow-up could not be calculated for 12 patients due to missing results of the last MAG-3 scan.

**Table 3:** Correlations between split kidney function and normalised length of the unaffected kidney, stratified for split kidney function

	*n*	*r*_s_	95% CI
*At presentation*			
Overall	92 a	0.18	(–0.01 to 0.37)
Split function <60%	67	0.13	(–0.08 to 0.35)
Split function ≥60%	25	0.08	(–0.38 to 0.52)
*At end of follow-up*			
vOverall	115 b	0.27	(0.08 to 0.44)
Split function <60%	81	0.02	(–0.19 to 0.23)
Split function ≥60%	34	0.47	(0.19 to 0.68)

CI = confidence interval; *n* = number; *r*_s_ = Spearman correlation coefficient.

a The correlation at presentation could not be calculated for four patients due to missing results of the first MAG-3 scan.

b The correlation at the end of follow-up could not be calculated for 12 patients due to missing results of the last MAG-3 scan.

When comparing children in the highest quartile of corrected kidney length to children in the lowest quartile (Table 4), the median split kidney function at the end of follow-up was higher in children in the highest quartile (OR 1.08, 95% CI 1.01–1.14, for every % increase in split kidney function). An elevated OR for having an enlarged kidney was also observed for a right-sided UPJO (OR 4.5, 95% CI 1.0–20). Lastly, we found a statistically significant correlation between age at last ultrasound and age-corrected kidney length (Table 4), indicating that an effect of older age remained present despite using an age-corrected kidney length.

**Table 4:** Crude and adjusted associations of several demographic and clinical characteristics comparing children with unilateral ureteropelvic junction obstruction and an unaffected kidney length in the highest quartile at the last follow-up with those in the lowest quartile

	Small kidney (Q1; *n* = 31)	Large kidney (Q4; *n* = 32)	cOR a	aOR (95% CI) b
Female gender	10 (32)	11 (34)	1.1 (0.4–3.1)	0.6 (0.2–2.4)
Right UPJO	5 (16)	11 (34)	2.7 (0.8–9.1)	**4.5 (1.0–20)**
Prematurity <37 wk c	1 (3.3)	3 (11)	3.5 (0.3–36)	3.0 (0.2–41)
Birth weight (g) d	3425 (2970–3910)	3400 (3215–3730)	1.00 (0.91–1.10)	1.02 (0.92–1.14) e
Any maternal medication use	3 (9.7)	2 (6.3)	0.6 (0.1–4.0)	0.8 (0.1–7.4)
Diagnosed >1 yr	13 (42)	20 (63)	2.3 (0.8–6.3)	1.6 (0.2–12)
Age at last ultrasound (yr)	4.1 (3.3–9.7)	8.0 (4.5–12)	**1.11 (1.00–1.24)**	1.11 (0.90–1.36) e
Split kidney function at last MAG3 scan (%) f	54 (50–56)	59 (52–77)	**1.08 (1.02–1.15)**	**1.08 (1.01–1.14)**^e^

aOR = adjusted odds ratio; CI = confidence interval; cOR = crude odds ratio; UPJO = ureteropelvic junction obstruction.

Data are presented as median (interquartile range) or *n* (%).

Odds rations in bold were statistically significant.

a Crude odds ratios were calculated using a binary logistic regression model with quartile of kidney length as the outcome and the respective factor as the determinant.

b Adjusted odds ratios were calculated using a binary logistic regression model with quartile of kidney length as the outcome and all other factors as determinants.

c Available in 30/31 patients with a small kidney and in 28/32 patients with a large kidney.

d Available in 29/31 patients with a small kidney and in 27/32 patients with a large kidney.

e Entered in the model as a continuous variable (per 100 g [birth weight], per year [age], and per % [% function]).

f Available in 26/31 patients with a small kidney and in 29/32 patients with a large kidney.

Signs of kidney injury were present in 68 children (35%), of whom 12 had an eGFR of <60 ml/min/1.73 m^2^, 43 showed high blood pressure, nine had proteinuria, and 16 used antihypertensive medication for one of these indications. The median age-corrected kidney length was 1.04 (interquartile range [IQR] 1.00–1.11) in children with and 1.05 (IQR 0.96–1.12) in children without kidney injury (*p* = 0.99). In the highest quartile of kidney length, nine children had signs of kidney injury versus eight in the lowest quartile (*p* = 0.84). None of the demographic and clinical characteristics were associated with the presence of kidney injury in multivariable logistic regression analyses (data not shown).

## 4 Discussion

In our study, we found that compensatory growth of the unaffected kidney is more common in children with a unilateral UPJO. A right-sided UPJO was the only other determinant that was associated with kidney length. Signs of kidney injury were found in ∼35% of patients, but no associations with kidney length were observed.

The correlation between split kidney function and kidney length was more clearly visible at the end of follow-up (median age 6.5 yr) than at presentation (median age 7 mo). Although this seems to be as expected, it is not in line with previous findings that showed a rapid increase in kidney parenchymal volume after nephrectomy and a stable length afterwards [16], [17]. It should be noted, however, that the patients in these studies were mostly adults, and physiological differences between children and adults may interact with the mechanisms that drive compensatory kidney growth.

Another interesting finding is that a correlation between split kidney function and kidney length is absent (*r*_s_ = 0.02) in children with split kidney function <60%, whereas there seems to be a correlation (*r* = 0.47) in children with split kidney function ≥60%. This is different from a study in adults, were a linear relationship was observed across the entire range of kidney mass reduction [6]. In another study, a strong correlation (*r* = 0.88) between kidney volume and function was observed in children with vesicoureteral reflux, even in children with split kidney function of 45–50% [18]. As we had data only on kidney length and not on volume, a different shape of the kidney could hypothetically explain the difference between this study and ours. However, previous studies reported strong correlations between kidney length and volume measurements, and visual inspection of the stored ultrasound images does not support this hypothesis [19], [20].

In our study, we also investigated whether other factors were associated with compensatory kidney hypertrophy. Even though we used age-corrected kidney length as an outcome variable, a correlation between age at last ultrasound and age-corrected kidney length remained present. This could indicate that compensatory kidney growth in children is a process that continues for several years. After inclusion of age at last ultrasound in our multivariable model, split kidney function and a right-sided UPJO were the only determinants of kidney hypertrophy. Many previous studies showed that, on average, the left kidney is bigger than the right kidney and it is also easier to measure due to its lower position [10], [21], [22], [23], [24], [25], [26]. Since the reference values used did not differentiate between sides of the kidney, we expected that having a right-sided UPJO results in a higher chance of having a larger than normal unaffected kidney. The difference between left and right kidney length was indeed more pronounced using normalised than unadjusted kidney lengths.

We found that 35% of the patients in our study showed signs of kidney injury, but we did not observe associations between signs of kidney injury and any of the determinants in our cohort, despite previously reported associations [27], [28]. Most likely, the absence of such associations is due to a lack of power in our study. In addition, kidney length could influence the prevalence of kidney injury both positively and negatively: a small or normal kidney length can be a reflection of a relatively mild UPJO, in which case no injury is expected, but can also indicate failed compensation. Similarly, a large kidney could indicate a severe UPJO, which by itself can cause hypertension or other signs of kidney injury, but can also reflect adequate compensation of the unaffected kidney. Larger cohorts with repeated measurements of both kidney length and kidney injury parameters are needed to provide more information on the prognostic value of kidney length. In addition, other biomarkers such as ultrasound renal parenchymal area, renal parenchymal thickness, or presence of specific genetic risk alleles should be investigated to determine the best strategy to identify children with a UPJO who are at risk of kidney injury as early as possible [19], [29], [30].

Our study has several limitations, some of which are inherent to a retrospective medical file review. Furthermore, our analysis may be hampered by including both children in whom UPJO was discovered prenatally and children who were diagnosed in early childhood because of urinary tract infections. Ultrasound scans were performed by different radiologists from the three participating hospitals. Therefore, interobserver variability could not be ruled out. To rule out differences among hospitals, we included the hospital as an independent variable in the logistic regression analyses, but this did not change the results substantially. Lastly, children were not followed according to a standardised follow-up protocol, but at the treating physician’s discretion. Therefore, more severely affected children could be over-represented in our study. This may have limited our possibility of finding determinants for compensatory hypertrophy and kidney injury. However, we do not expect that this limitation changed the observed correlations between split kidney function and kidney length.

## 5 Conclusions

In conclusion, we showed that compensatory hypertrophy seemed to be present in most children with a unilateral reduction of kidney function after sufficient time of follow-up. Furthermore, a correlation between split kidney function and kidney length was observed in children with a more severe UPJO. Contralateral kidney length provided no clear prognostic value for developing kidney injury. Therefore, studies with a larger number of patients, additional potential biomarkers of kidney injury, and longer follow-up duration are needed to examine prognostic factors for developing kidney injury in UPJO patients. If such prognostic factors are found, management of children with a unilateral UPJO can be personalised further.

  ***Author contributions:*** Sander Groen in ’t Woud had full access to all the data in the study and takes responsibility for the integrity of the data and the accuracy of the data analysis.

  *Study concept and design*: Groen in ’t Woud, Schreuder, van der Zanden.

*Acquisition of data*: Quaedackers, Nijman, Steffens, de Wall, van der Zanden.

*Analysis and interpretation of data*: Groen in ’t Woud, Reuver.

*Drafting of the manuscript*: Groen in ’t Woud, Reuver.

*Critical revision of the manuscript for important intellectual content*: Feitz, Quaedackers, Nijman, Steffens, de Wall, Roeleveld, Schreuder, van der Zanden.

*Statistical analysis*: Groen in ’t Woud, Reuver.

*Obtaining funding*: Feitz, Roeleveld, Schreuder, van der Zanden.

*Administrative, technical, or material support*: None.

*Supervision*: Feitz, Roeleveld, Schreuder, van der Zanden.

*Other*: None.

  ***Financial disclosures:*** Sander Groen in ’t Woud certifies that all conflicts of interest, including specific financial interests and relationships and affiliations relevant to the subject matter or materials discussed in the manuscript (eg, employment/affiliation, grants or funding, consultancies, honoraria, stock ownership or options, expert testimony, royalties, or patents filed, received, or pending), are the following: Loes van der Zanden is supported by a VENI grant from the Dutch Research Council (NWO; 91618036) and a Kolff junior postdoc grant from the Dutch Kidney Foundation (13OKJ36).

  ***Funding/Support and role of the sponsor:*** None.
